# Peripheral Nerve Block Practice in Portugal: A National Survey

**DOI:** 10.7759/cureus.35478

**Published:** 2023-02-26

**Authors:** Custódia Teixeira, Vanessa Artilheiro, Ronald Silva, Marta Pereira, Joana Magalhães

**Affiliations:** 1 Anesthesiology, Centro Hospitalar Universitário de Lisboa Central, Lisbon, PRT; 2 Anesthesiology, Centro Hospitalar de Lisboa Ocidental, Lisbon, PRT; 3 Anesthesiology, Hospital da Senhora da Oliveira, Guimarães, PRT

**Keywords:** training & education, ultrasonography, regional anaesthesia, peripheral nerve block, portugal, survey

## Abstract

Regional anesthesia (RA) has several benefits and its use has increased with the advent of ultrasound-guided techniques. Opioid-sparing anesthesia and reduced use of general anesthesia are some of the mainstay advantages of RA. Although anesthetic practices differ deeply between countries, RA has assumed a crucial role in the daily practice of anesthesiologists, particularly during the COVID-19 pandemic period.

This cross-sectional study provides an overview of peripheral nerve block (PNB) techniques performed in Portuguese hospitals. An online survey was reviewed by members of Clube de Anestesia Regional (CAR/ESRA Portugal) and then sent to a national mailing list of anesthesiologists. The survey focused on specific topics related to RA techniques such as the importance of training and experience, and the relevance of logistical limitations during the execution of RA. All data were collected anonymously and included in a Microsoft Excel (Microsoft Corp., Redmond, WA, USA) database, for further analysis. A total of 335 valid answers were obtained.

All participants considered RA as a key competence in their daily practice. Half of those inquired performed PNB techniques once to twice per week. The main limitations identified for performing RA in Portuguese hospitals were the absence of block rooms and insufficiently trained personnel for the appropriate and safe execution of these techniques.

This survey provides a comprehensive overview of RA in the Portuguese setting and could serve as a baseline for further studies.

## Introduction

Currently, peripheral nerve blocks (PNBs) play a key role in the perioperative setting, either alone or combined with other anesthetic techniques. Performing PNBs enables anesthesia without airway manipulation and adequate acute pain management, whilst minimizing opioid consumption and improving postoperative recovery. Also, these techniques reduce the risk of postoperative pulmonary complications, nausea and vomiting, cognitive dysfunction, and delirium [1]. The emergence of new challenges during the COVID-19 pandemic has highlighted the central role of regional anesthesia (RA) in the daily practice of anesthesia, as it avoids aerosol-generating procedures and improves safety in the operating theatre for patients and healthcare personnel [2].

This focus on RA provided a growing interest in survey implementation to provide insights into anesthetic practices worldwide, including variations between hospitals within the same country. Some nationwide surveys are documented in the literature, with questionnaires designed to get an insight into PNB techniques [3-5]. A progressive effort to submit validated questionnaires is ongoing; however, further advances in the literature still need to be pursued to implement international standardized surveys [6].

Therefore, this specific survey aims to investigate the role of PNB techniques performed in the national hospitals of Portugal, as well as the knowledge, education programs, and accessibility of training related to RA practices. The study stands out as the first survey applied in Portugal, fully supported by a RA national society, the Clube de Anestesia Regional (CAR/ESRA Portugal).

## Materials and methods

We created a questionnaire comprising 21 questions to assess RA practices in Portugal. The questionnaire was reviewed by CAR/ESRA members and distributed to all Portuguese anesthesiologists (consultants and residents) on the CAR/ESRA society mailing list (n=2504) via email, using an online form. A representative sample of 334 individuals was required to achieve a confidence level of 95%.

Voluntary responses were collected, and anonymity was guaranteed. Data collection took place between July 25 and October 31, 2021. Of the 21 questions, 20 were closed-ended, and one was open-ended. Only 14 questions were subjected to statistical evaluation (Table 1). The remaining questions were excluded as they did not align with the study's objectives.

**Table 1:** Questionnaire structure Listed above are the 14 closed-ended questions that were subjected to statistical analysis. PNB: Peripheral nerve block

Questionnaire	
1	Gender
2	Age
3	Hospital
4	Institution
5	Is regional anesthesia a fundamental skill?
6	How many PNBs do you perform on average, each week?
7	In which surgical specialties do you perform most PNBs?
8	Which PNBs do you consider essential for anesthesiology practice? (choose 5 from the list)
9	Which technique do you use for nerve localization?
10	Which advantages do you see in ultrasound use?
11	Under which of the following clinical scenarios, do you not perform PNBs?
12	Which aseptic techniques do you use when performing a single-shot PNB?
13	Which complications have you experienced when performing PNBs?
14	What barriers to PNB practices have you observed in your hospital?

The primary objective of the study was to evaluate RA practices in Portugal. Secondary objectives included determining the importance of RA among Portuguese anesthesiologists, namely the essential PNBs for most surgeries, and the generalized limitations to its practice. We analyzed the collected data using Microsoft Excel (Microsoft Corp., Redmond, WA, USA) and results were presented as absolute numbers and percentages.

This study was conducted per the principles of the Declaration of Helsinki and the approval of the Ethics Committee for Health and the Scientific Research for Health of the Hospital da Senhora da Oliveira, Guimarães (approval no. 03/2023).

## Results

The questionnaire was answered anonymously and voluntarily by 335 individuals, of whom 70% were female, and 69% were between the ages of 31 and 51 (Table 2). Most anaesthesiologists worked in the public health system, from the North and Lisbon areas (Table 3). 

**Table 2:** Demographics of the participants The participants' demographic characteristics have been organized by gender, age groups, and healthcare sectors.

Demographics	number (n)	%
Gender		
Female	234	69.85%
Male	101	30.15%
Age (years)		
≤30	36	10.75%
31-40	157	46.87%
41-50	75	22.39%
51-60	41	12.24%
≥60	26	7.76%
Healthcare System		
Private	41	12.24%
Public	294	87.76%

**Table 3:** Geographical distribution of participants The geographical distribution of participants has been organized by health regions (North, Center, Lisbon and Tagus Valley, Alentejo and the Algarve) and autonomous regions (Azores and Madeira).

Region	number (n)
Lisbon and Tagus Valley	130
North	127
Center	53
Alentejo	11
Algarve	6
Autonomous Region of Madeira	6
Autonomous Region of the Azores	2
Total	335

Competency in RA was evaluated using a ten-point scale. Around 62% of participants considered it as fundamental, assigning the maximum score (Table 4). Almost half of the inquired anesthesiologists (40%) performed PNBs once to twice per week (Table 5), mainly in orthopedics, general surgery, and plastic surgery. 

**Table 4:** Survey results to the question "Is regional anesthesia a fundamental skill?" Competency in RA was evaluated using a ten-point scale, the options started with "strongly disagree" at 1 and finished with "strongly agree" at 10. None of the respondents answered below 5.

Rating scale	Number (n)	%
<5	0	0%
5	3	0.90%
6	8	2.39%
7	11	3.28%
8	41	12.24%
9	64	19.10%
10 (Strongly agree)	208	62.09%

**Table 5:** Average number of PNBs per week All the participants answered a range of PNBs were executed per week, on average. PNB: Peripheral nerve block

Number of PNBs per week	Number (n)	%
0	37	11.04%
1-2	132	39.40%
3-5	88	26.27%
6-9	44	13.13%
≥10	34	10.15%

**Figure 1:**
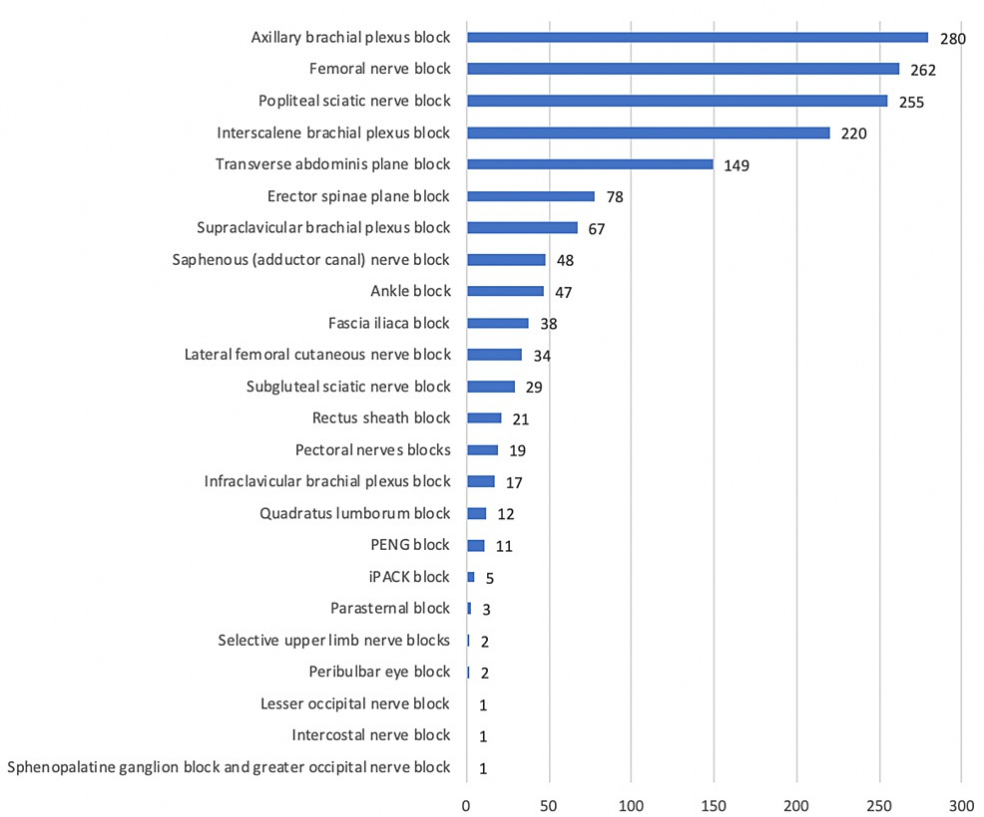
Most important PNBs The respondents chose five nerve blocks they considered essential, from the options featured in the above figure. We had a unanimous agreement on the five most essential PNBs PNB: Peripheral nerve block, PENG: Pericapsular nerve group, iPACK: Infiltration between popliteal artery and capsule of the knee

The PNBs considered as “the top 5 blocks every anaesthesiologist should perform” were: brachial plexus block (axillary and interscalene approach), femoral nerve block, sciatic nerve block (popliteal approach), and transversus abdominis plane block, as presented in Figure 1.

**Figure 2:**
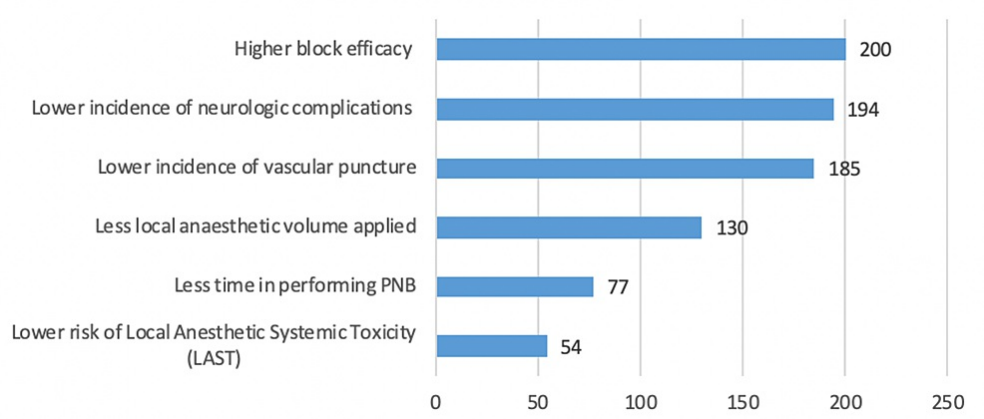
Perceived advantages of ultrasound use The participants chose the advantages of ultrasound use from a specified list. Each participant could only select a maximum of three options. PNB: Peripheral nerve block

Ultrasonography (US) alone was the most frequently used guidance tool for nerve localization (75.2%). The main advantages of US use were higher block efficacy, lower incidence of neurologic complications, and lower incidence of vascular puncture (Figure 2). Participants reported performing an aseptic technique for single-shot PNBs, besides not using a sterile gown and drape (Table 6). 

**Table 6:** Equipment used to perform aseptic PNB technique The question was directed at the aseptic conditions used to perform single-shot PNBs, and allowed multiple choices. PNB: Peripheral nerve block

Equipment		number (n)
Skin disinfection		320
Sterile gloves		258
Sterile gel		226
Sterile ultrasound cover		178
Non-sterile ultrasound cover		85
Non-sterile gloves		62
Non-sterile gel		48
Sterile gown		24
Added options by part	Sterile drape	1
	Total aseptic techniques in neuraxial blocks and continuous PNBs	2

**Figure 3:**
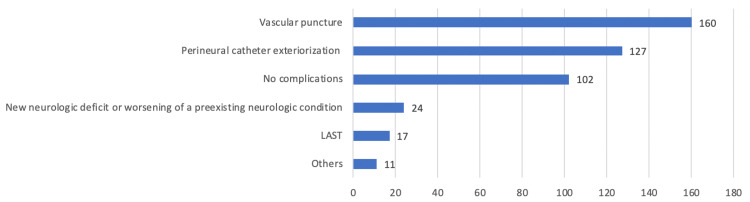
Most frequently observed complications The participants could choose multiple options and add other complications not included in the answers. The complications added were: incomplete block (8 participants), seizure without intravascular administration (1 participant), pneumothorax (1 participant), and transient Horner syndrome (1 participant). LAST: Local anesthetic systemic toxicity

The most common complications reported were vascular puncture and perineural catheter dislodgement. Around 7.2% also reported neurological deficits and other nerve-related complications (Figure 3). The majority of respondents did not perform PNBs in cases of local infection, high-risk of neurological damage or complications during or before the surgical procedure, patients on antithrombotic drugs without the correct suspension, and risk of compartment syndrome. Specific clinical scenarios are detailed in Table 7.

**Table 7:** Specific situations where PNBs are not performed The respondents answered based on a list of specific situations (options listed above), and add other options. The options added were absolute contraindications to perform PNBs. PNB: Peripheral nerve block

Under which of the following clinical scenarios do you not perform PNB?	Number (n)
Local infection	311
High-risk of neurological compromise during surgery	221
Insufficient time suspension of antithrombotic drugs	218
High-risk of compartment syndrome	217
Preexisting neurological deficits	200
Sepsis	145
Performing PNB after neuroaxial anesthesia	30
Performing PNB after general anesthesia	17
Other	17

Only 22% of respondents reported no limitations in performing PNBs. The absence of a block room (45.37%), lack of skills/training and provider's inexperience (64.78%), and the possible delays in operating theatre (34.63%) were reported as the main reasons for not performing PNBs (Table 8).

**Table 8:** Barriers to performing PNBs Multiple options were accepted in this question. PNB: Peripheral nerve block

What barriers to PNB practices have you observed in your hospital?	Number (n)
Absence of block room	152
Provider's inexperience	130
Operating theatre delays	116
Lack of skills or training	87
Disagreement between anaesthesiologists and surgeons about potential benefits	57
Absence of Acute Pain Service for postoperative evaluation	54
Lack of nursing staff training	48
Lack of ultrasound machine	35
Lack of material (needles, catheters)	34
No limitations	75

## Discussion

Regional techniques are invaluable tools for all anesthesiologists, particularly in life-threatening scenarios, with an impact on global patient outcomes [1]. Peripheral nerve blocks are a relatively recent advance in RA, and the majority of the survey participants were anesthesiologists aged 31 to 50 years old, reflecting a specific bias in the results. Also, this study was mostly validated in the largest metropolitan areas, which may reflect a higher differentiation level and resource availability. The participants highlighted the lack of opportunity to perform PNBs, with 40% of all anesthesiologists performing only one to two blocks per week, particularly in orthopedics, general surgery, and plastic surgery. As supported by a questionnaire sent to all German anesthesia departments, large hospitals provided more advanced regional techniques, which is also corroborated by this survey since most professionals from Lisbon and Oporto perform a large number of PNBs [7].

This survey unveiled the top five core blocks from the participating anesthesiologists: brachial plexus block (axillary and interscalene approach), femoral nerve block, sciatic nerve block (popliteal approach), and transversus abdominis plane block. These data are consistent with a survey of all anesthesiologists who graduated from the two major Chilean residency training anesthesia programs [8]. These basic PNBs cover most areas of an important number of surgical procedures, enhancing the role of high-value basic ultrasound-guided RA techniques. Every anesthesiologist should have competence in “Plan A” blocks: brachial plexus (axillary and interscalene approach), femoral nerve and adductor canal block, sciatic nerve block (popliteal approach), rectus sheath block (abdominal wall), and erectors spinae block (thoracic wall) [9].

Concerning nerve localization techniques, most of the participant anaesthesiologists (75.2%) performed US-guided PNBs, as opposed to dual-guidance, nerve stimulation, or anatomical landmark techniques. These participants stated clear advantages to the US-guided technique: higher block efficacy and safety, real-time visualization of anatomic goal structures, and better needle-nerve image control, and reduced risk of accidental vascular puncture. The European Society of Anaesthesiology and Intensive Care (ESAIC) recently published recommendations on the perioperative use of US (PERSEUS-RA), highlighting the advantages of US-guided PNBs, as supported by other studies: less time to perform the block, higher comfort, and patient satisfaction related to less needle repositioning and lower volume of local anesthetic required, with reduced risk of local anesthetic systemic toxicity (LAST) [10]. However, these advantages depend on the patient’s sonoanatomy and the specific anatomical area to place the nerve block. There is still no data supporting the advantages of US-guided PNB and neurological injury concerning other nerve localization methods, as the incidence of nerve injury is usually multifactorial [11,12].

On the other hand, RA is not without risks and complications, whether related to single-shot or continuous PNB. The insertion of a perineural catheter imposes infection control practices, but for single-shot blocks, a standardized approach is still a work in progress. Most US-guided single-injection PNBs have a low infection rate, using only regular disinfection techniques and a sterile barrier dressing for the transducer [13]. Several recommendations include aseptic measures such as the use of sterile gel and a sterile transducer cover, in addition to the use of sterile gloves, face mask, and adequate skin disinfection [14]. These suggestions are consistent with our results as most anesthesiologists follow the same aseptic recommendations.

Fortunately, complications associated with PNBs are rare events, consistent with the fact that nearly one-third (30%) of the surveyed anesthesiologists reported no complications to date. However, accidental vascular puncture and inadvertent perineural catheter removal were identified as the most frequent complications. The literature describes these complications as not uncommon, with reported incidences of vascular puncture and hematoma formation ranging from 5.7% to 6.6%, and inadvertent catheter removal from 1% to 1.4% [15].

Neurological deficits are one of the most severe complications that can result from PNBs. Nevertheless, permanent nerve injury is rare and certain measures can be taken to prevent post-block neurological symptoms, such as clinical monitoring, a tangential approach to the nerve, dual guidance for nerve localization, a lower concentration of local anesthetic, and injection pressure monitoring. Despite being considered good practices for performing PNBs, the literature shows inconsistent data regarding nerve damage associated with some of these measures [16].

Although PNBs have a valuable impact, specific criteria should be taken into account when performing regional techniques. According to most participants, PNBs should be avoided in the case of local or systemic infection [17], patients taking antithrombotic drugs [18], high surgical risk of nerve injury, documented or known neurological disease, and risk of acute compartment syndrome [19]. Given the lack of consensus regarding the relative contraindications of RA, anesthesiologists must consider performing PNBs on a case-by-case basis.

One of the objectives of this survey was to obtain a comprehensive and realistic view of the limitations faced by each hospital in the performance of PNBs. The survey identified the lack of a dedicated block room, the lack of skills/training, and provider's inexperience, and delays in operating theatre as the primary barriers that hinder the widespread use of RA. Providing adequate resources for performing PNBs in a well-equipped block room not only enhances theater efficiency but also facilitates education and training in this field [20].

This survey gathered data from 335 anesthesiologists via a CAR/ESRA mailing list. However, one limitation of this method is the self-selection bias of participants, as those who chose to respond may have a particular interest in RA compared to those who did not participate. To mitigate this limitation, a valid alternative would be to randomly select participants by directly contacting institutions and anesthesia departments.

## Conclusions

Regional anesthesia has played a crucial role in managing challenging scenarios, including the COVID-19 pandemic, and is widely recognized as a major skill among the anesthetic community. Our study reveals that PNB techniques are mostly performed by graduated anesthesiologists aged 31 to 50, particularly in the North and South regions of Portugal. However, these anesthesiologists typically perform PNB techniques only once or twice per week. This is due to several factors, including the absence of block rooms, insufficiently trained personnel to perform these techniques accurately, and the possible start time delays in the operating theatre.

Another noteworthy finding is the consistency among the top 5 core blocks considered by the surveyed anesthesiologists. Efforts should be made to educate the anesthesiology community about the "Plan A" blocks.

To sum up, this survey provides a comprehensive overview of the daily practice of anesthesiologists in Portugal, with a particular focus on PNBs. The findings of this study may serve as a valuable baseline for future surveys and comparative studies on practices abroad.
